# Posterior Single-Direction Approach for Thoracoscopic Combined S2+S6 Segmentectomy in Fused Fissure: A Case Report

**DOI:** 10.70352/scrj.cr.25-0559

**Published:** 2026-02-03

**Authors:** Yasuhiro Nakashima, Hirotomo Takahara, Ayaka Asakawa, Ryo Wakejima, Hironori Ishibashi

**Affiliations:** 1 Department of Thoracic Surgery, Tokyo Kyosai Hospital, 2-3-8 Nakameguro, Meguro-ku, Tokyo 153-8934, Japan; 2 Department of Thoracic Surgery, Institute of Science Tokyo, 1-5-45 Yushima, Bunkyo-ku, Tokyo 113-8519, Japan

**Keywords:** pulmonary segmentectomy, fused fissure, single-direction approach, hilar first, lung neoplasms

## Abstract

**INTRODUCTION:**

Segmentectomy in cases with fused fissures poses technical challenges due to increased risks of air leakage, as reported in lobectomy. The single-direction approach avoids fissure manipulation through hilar-first dissection but has been rarely reported for posterior segments. We report the first case of a posterior single-direction approach for combined S2+S6 segmentectomy in a patient with a tumor located in an incomplete right interlobar fissure.

**CASE PRESENTATION:**

A 52-year-old woman with bilateral multiple ground-glass nodules presented with a 22-mm heterogeneous ground-glass nodule located in an incomplete fissure between the right upper and lower lobes. Preoperative CT revealed a fused fissure and concomitant S2 lesions, including a 15-mm subpleural nodule and a 7-mm deeper parenchymal nodule, as well as a 7-mm S6 lesion. Three-port thoracoscopic S2+S6 segmentectomy was performed with the surgeon positioned dorsally and ports placed posteriorly. Selective segmental inflation was performed prior to hilar dissection to mark intersegmental planes. Sequential division of V6 and the common trunk of ascending A2 and A6 was performed from the posterior hilar approach, followed by B6 division using a suction-device trick. After intersegmental division of S6 to improve hilar exposure, B2 was divided using the same approach, followed by V2 division and completion of S2+S6 segmentectomy. The postoperative course was uneventful.

**CONCLUSIONS:**

The posterior single-direction approach successfully achieved complete oncologic resection without fissure manipulation, providing a safe alternative for posterior segmentectomy in patients with fused fissures.

## Abbreviations

GGN
: ground-glass nodule
VATS
: video-assisted thoracoscopic surgery

## INTRODUCTION

Segmentectomy has emerged as an effective surgical option for early-stage non-small cell lung cancer in specific subgroups.^1,2)^ In cases with fused fissures, the traditional interlobar fissure approach raises concerns of increased morbidity risks including air leakage, as reported for lobectomy.^3,4)^ The single-direction approach, avoiding fissure manipulation by dissecting hilar structures in advance, has been developed as an alternative method for segmentectomy, adapting the fissureless technique from lobectomy.^5)^ However, the single-direction approach for posterior segments presents technical challenges,^6)^ and clinical experience remains limited. We report the first case of a single-direction approach from the posterior side for combined S2+S6 segmentectomy in a patient with a tumor located in an incomplete right upper-lower interlobar fissure.

## CASE PRESENTATION

**Fig. 1:**
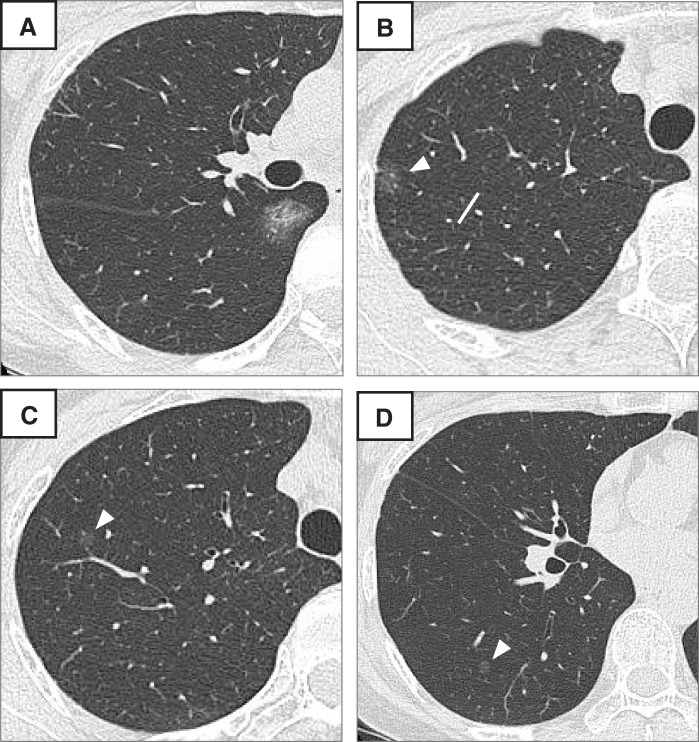
Preoperative CT images. (**A**) Axial CT showing a 22-mm part-solid ground-glass nodule located in the incomplete fissure between the right upper and lower lobes, with dense parenchymal fusion. (**B**) Axial image showing a 15-mm pure ground-glass nodule in the S2 segment (white arrowhead). (**C**) Axial image showing a 7-mm pure ground-glass nodule in the S2 segment (white arrowhead). (**D**) Axial image showing a 7-mm pure ground-glass nodule in the S6 segment (white arrowhead).

**Fig. 2:**
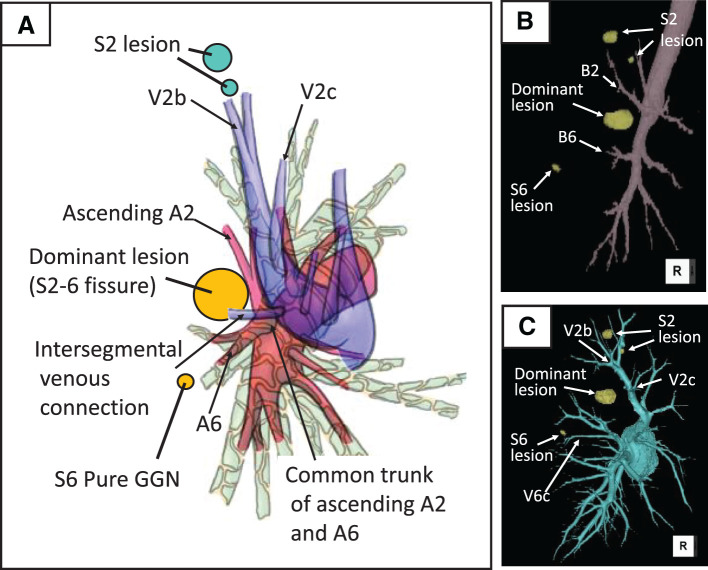
Anatomical relationships of lesions and hilar structures. (**A**) Schematic illustration of bronchial and vascular anatomy based on CT, showing hilar vascular anomalies including a common trunk of ascending A2 (serving as the common branch for both A2a and A2b) and A6, and intersegmental venous connection from S6 to the central vein. All 4 lesions (dominant lesion in the fissure, 2 S2 lesions, and 1 S6 lesion) are depicted. (**B**) 3D reconstruction of the right bronchial tree showing the close proximity of the dominant lesion to both B2 and B6 segmental bronchi, with the locations of all 4 lesions indicated. (**C**) 3D reconstruction of the right pulmonary veins showing the 2 S2 lesions located near V2b (intersegmental vein between S2a and S2b) with V2c serving as an intersegmental landmark, and the S6 lesion positioned slightly cranial to V6c with V6c serving as an intersegmental landmark.

A 52-year-old asymptomatic woman with a history of right breast cancer presented with bilateral multiple ground-glass nodules (GGNs) on routine follow-up CT. The patient had a past smoking history. She demonstrated normal pulmonary function and adequate surgical tolerance. The dominant lesion was a 22-mm heterogeneous GGN with a 2-mm solid component located in the incomplete fissure area between the right upper and lower lobes, suggestive of primary lung cancer (cT1aN0M0, Stage IA1) (**Fig. 1A**). Additional ipsilateral lesions included a 15-mm pure GGN (**Fig. 1B**) and a 7-mm pure GGN (**Fig. 1C**) in the S2 segment, a 7-mm pure GGN in the S6 segment (**Fig. 1D**), and a 12-mm pure GGN with barely perceptible density in the middle lobe. Contralateral lesions consisted of a 7-mm part-solid GGN with a 1-mm solid component and an 8-mm pure GGN in the left upper lobe. PET revealed a standardized uptake value maximum of 1.4 in the dominant lesion, while other nodules showed no significant uptake. The dominant lesion was positioned adjacent to the B2 and B6 bronchi, and dense parenchymal fusion was noted at the right oblique fissure (**Figs. 1A** and **2A**, **2B**). Hilar vascular anomalies included a common trunk of ascending A2 (serving as the common branch for both A2a and A2b) and A6, as well as an intersegmental venous connection from S6 to the central vein running between these vessels (**Fig. 2A**). Detailed anatomical relationships between the lesions and bronchial/vascular structures are demonstrated in **Supplementary Video 1**.

The treatment plan included combined S2+S6 segmentectomy to address the dominant lesion and multiple pure GGN lesions in the S2 and S6 segments. The dominant lesion in the fissure was positioned within 10 mm of both B2 and B6 segmental bronchi (**Fig. 2B**), requiring division of both bronchi to secure adequate resection margins. The 15-mm S2 lesion was subpleural, whereas the 7-mm S2 lesion lay deeper within the parenchyma (approximately 25 mm from the pleural surface) (**Fig. 1C**). Wedge resection would have been unable to secure adequate margins for both lesions simultaneously. Preoperative planning confirmed that all S2 lesions (located near V2b) and the S6 lesion (cranial to V6c) would be encompassed within the resection margins by creating intersegmental planes along V2c and slightly caudal to V6c, respectively (**Fig. 2C**). The middle lobe and contralateral lesions were designated for CT surveillance given their small size and predominantly ground-glass characteristics, with re-evaluation planned at 6-month intervals. A posterior single-direction approach was selected due to the impalpable tumor’s challenging location within the dense fissure and to minimize manipulation around the middle lobe hilum, preserving surgical options for potential staged resection if progression was observed in the conservatively managed lesions.

The procedure was performed under general anesthesia with 1-lung ventilation in the left lateral decubitus position using a 3-port completely thoracoscopic approach, with the surgeon standing on the dorsal side (**Supplementary Video 2**). Carefully preserving the long thoracic nerve, port placement included a 3.5-cm access port and a 5-mm camera port, both positioned at the midaxillary line at the sixth and eighth intercostal spaces, respectively, and an 11.5-mm assistant port at the sixth intercostal space on the anterior axillary line. The surgeon primarily performed tissue dissection using curved forceps and a bipolar vessel-sealing device, while staplers were inserted through the access port. A 5-mm angled rigid scope was inserted through the camera port.

Intraoperative findings confirmed a fused fissure. None of the lesions demonstrated pleural changes, and all were impalpable. Initially, selective bronchial inflation was performed to identify intersegmental planes using a flexible bronchoscope. The bronchoscopist selected the target subsegmental bronchi (B6b, B6c, B2a, and B2b) and performed wedging at these levels, then manually insufflated air using a 50-mL syringe. After approximately 10 insufflations, adequate subsegmental expansion was achieved, and the intersegmental planes were marked with electrocautery and sutures.

**Fig. 3:**
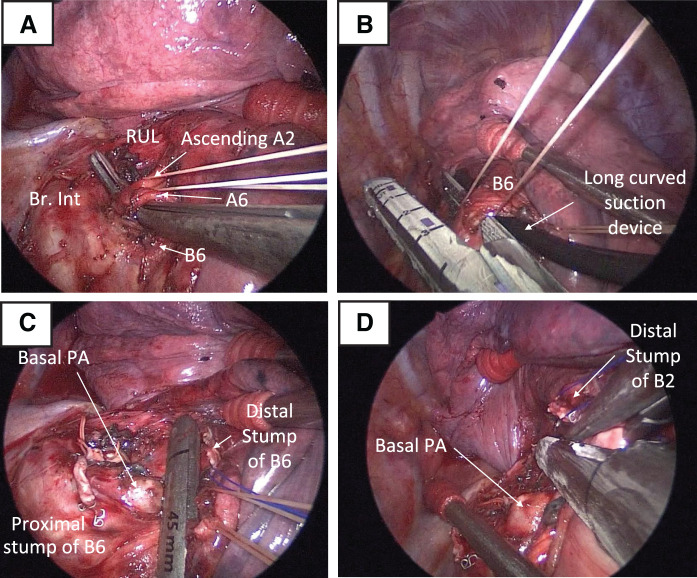
Intraoperative findings. (**A**) Hilar dissection from the posterior approach showing an isolated common trunk of ascending A2 and A6. (**B**) Suction device trick for safe B6 division with curved suction. Intersegmental plane division between S6-basal segments (**C**) and between S2 and S1, S3 segments (**D**). Br.Int, bronchus intermedius; PA, pulmonary artery; RUL, right upper lobe bronchus

Hilar dissection was initiated from the posterior approach, and V6 and the common trunk of ascending A2 and A6 were sequentially divided (**Fig. 3A**). After confirming the B6 anatomical position with bronchoscopy, a curved suction device was inserted ventral to B6 as a guide for stapler placement, allowing safe bronchial division (suction-device trick) (**Fig. 3B**).^7)^ The B6 peripheral stump was retracted with sutures to create additional hilar exposure, enabling identification and division of the intersegmental venous connection from S6 to the central vein. Subsequently, S6-basal intersegmental plane creation was performed using a stapler while retracting the bronchial and vascular stumps to maintain adequate working space (**Fig. 3C**). Following similar anatomical confirmation with bronchoscopy, B2 was divided using the same suction-guided technique. The B2 peripheral stump was retracted with sutures, allowing access to divide V2 in the deep hilar region. The intersegmental plane between S2 and S1, S3 was created continuously from the caudal S6-basal intersegmental plane toward the cranial direction (**Fig. 3D**), and the specimen was extracted.

The operative time was 249 min with minimal blood loss (10 mL). Although no postoperative air leak was observed, pleural effusion of 300 mL/day was noted on PODs 1–2, requiring chest tube removal on POD 4 after effusion reduction. The patient was discharged on POD 7. Pathological examination revealed that the dominant lesion (S2–6 fissure) was adenocarcinoma (pT1miN0M0; tumor size 15 mm, invasive size 1 mm, surgical margin 10 mm). The 15-mm S2 lesion was adenocarcinoma *in situ* (pTis; tumor size 8 mm, surgical margin 14 mm). The additional small 7-mm pure GGNs in S2 and S6 proved to be chronic inflammatory scarring tissue with 3-mm surgical margins.

## DISCUSSION

This case represents the first report of a single-direction approach from the posterior side for combined S2+S6 segmentectomy for tumors located in incomplete fissures. The posterior single-direction approach is defined by performing hilar dissection exclusively from the posterior aspect without fissure manipulation, with the dissection proceeding in a unidirectional manner from posterior to anterior. The visualization difficulties and exposure challenges of the interlobar pulmonary artery have been overcome by placing the main access port at the midaxillary line and avoiding fissure manipulation. This method establishes a useful surgical option for posterior segmentectomy in cases with fused fissures.

For segmentectomy in fused fissure cases, 2 principal approaches exist to avoid interlobar manipulation: the fissure-first method and the hilar-first approach, known as the single-direction approach.^6,8–10)^ The fissure-first approach is inapplicable for tumors located within incomplete fissures and requires extensive initial interlobar fissure dissection beyond the intended segmental resection area, resulting in excessive interlobar pulmonary artery exposure. In contrast, the single-direction approach remains applicable for interlobar tumor segmentectomy. Furthermore, it prevents the risk of tumor spillage during fissure manipulation in the conventional approach.

The single-direction approach has been reported for anterior and basal segments,^6,9,10)^ but reports for posterior segments remain limited.^11)^ Previously reported combined S2+S6 segmentectomies were performed via fissure dissection and posterior tunneling for intersegmental plane creation.^12,13)^ (**Table 1**) In most video-assisted thoracoscopic surgery (VATS) procedures, the main port is positioned ventrally at the anterior axillary line. This conventional port placement necessitates manipulation across the lung parenchyma to access the posterior hilum, leading to compromised visualization and instrument interference. For this reason, fissure-based approaches are commonly selected even in the presence of incomplete fissures. Furthermore, in fissure-based approaches, the stapler hinge is positioned near the tumor during initial intersegmental plane creation, whereas our approach positions the stapler tip near the tumor, providing improved control and visualization for margin assessment.

**Table 1:** Comparison of reported combined S2+S6 segmentectomy techniques

Feature	Han et al. (2017)	Gonzalez-Rivas et al. (2017)	Present case
Segments resected	S2+S6	S2+S6+S10a	S2+S6
Ports	Uniportal	Uniportal	Three port
Fissure status	Incomplete	Incomplete	Fused
Fissure dissection	Performed	Performed	None
Approach direction	Anterior (fissure) to posterior	Anterior (fissure) to posterior	Posterior to anterior
Pulmonary artery exposure	Via fissure dissection	Via fissure dissection	Posterior hilum direct access
Tunneling for intersegmental stapling	Required (fissure to posterior)	Required (fissure to posterior)	Not required
Key technical feature	First combined S2+S6 report	Extensive tri-segmental resection	Posterior single-direction approach

In this case, placement of the main port on the midaxillary line and the camera port positioned caudally, with dorsal surgeon positioning, ensured excellent posterior visualization. The lateral approach using grasping techniques from the midaxillary line was adapted from the concept of a lateral approach for uniportal lung resection.^14)^ Additionally, placing the assistant port ventrally within the same intercostal space as the main port avoided instrument interference, achieving safe and efficient access to the posterior segment hilum. The same-intercostal sub-port placement for enhanced instrument mobility and improved ergonomics was inspired by the modified VATS approach.^15)^

Technical challenges in the posterior single-direction approach include unfamiliar posterior-to-anterior visualization compared to conventional approaches, poor accessibility to deep hilar structures, and limited working space during segmental bronchial stapling due to vessels behind the bronchi. These challenges were addressed through detailed preoperative CT planning, strategic retraction of divided vascular and bronchial stumps with sutures, and the suction-device trick.^7)^ The suction-device trick enables safe bronchial division by protecting vessels with the suction device and providing stapler insertion space through retraction of the target bronchus.^7)^

In this case, intersegmental plane marking was performed before hilar processing. This prior marking approach enabled stapling under lung collapse conditions, providing excellent visualization and facilitating parenchymal retraction. During deep hilar dissection in single-direction approaches, partial preliminary division of intersegmental planes may be necessary to improve operative maneuverability. Prior marking allows continuation of deep dissection after intersegmental division without interference from the expanded lung. S6 intersegmental division before S2 processing improved visualization of the central vein area. For selective segmental inflation, staged inflation by manual jet ventilation offered adjustable insufflation control compared to high-frequency jet ventilation,^16)^ and bronchial wedging at the subsegmental level minimized expansion of adjacent segments. Although this approach successfully achieved negative margins for all lesions, the surgical margins of the small peripheral pure GGN lesions were limited to 3 mm. However, these lesions were pathologically confirmed as chronic inflammatory scar tissue, making these margins oncologically adequate. The adenocarcinoma *in situ* and minimally invasive adenocarcinoma achieved sufficient margins of 14 and 10 mm, respectively. For these impalpable lesions, combining anatomical landmarks with bronchoscopic or CT-guided marking techniques may enhance surgical confidence and margin assurance.

This study has several limitations. As a single-center case report, evaluation of anatomical variations, standardization, and reproducibility of the technique remains insufficient. The unidirectional approach, proposed as an extension of the single-direction approach concept to uniportal segmentectomy, has not been reported for its application to posterior segments.^6)^ Although we selected a multiport approach due to the anticipated longer operative time given our current experience, all staplers were inserted through the main port, suggesting potential applicability for future uniportal procedures.

## CONCLUSIONS

We successfully performed thoracoscopic right S2+S6 segmentectomy using a posterior single-direction approach for tumors located in an incomplete fissure. Strategic port placement and preoperative planning enabled efficient hilar access while avoiding fissure manipulation, resulting in no postoperative air leakage.

## SUPPLEMENTARY INFORMATION

Supplementary Video 1 Preoperative imaging assessment. Axial and sagittal CT images followed by 3D reconstruction with rotation view, demonstrating the spatial relationships between the lesions and hilar structures.

Supplementary Video 2 Thoracoscopic right S2+S6 segmentectomy using posterior single-direction approach.

## DECLARATIONS

### Availability of data and materials

The datasets supporting the conclusions of this article are included in **Figs. 1**–**3**.

## References

[1] Saji H, Okada M, Tsuboi M, et al. Segmentectomy versus lobectomy in small-sized peripheral non-small-cell lung cancer (JCOG0802/WJOG4607L): a multicentre, open-label, phase 3, randomised, controlled, non-inferiority trial. Lancet 2022; 399: 1607–17. 35461558 10.1016/S0140-6736(21)02333-3

[2] Aokage K, Suzuki K, Saji H, et al. Segmentectomy for ground-glass-dominant lung cancer with a tumour diameter of 3 cm or less including ground-glass opacity (JCOG1211): a multicentre, single-arm, confirmatory, phase **3** trial. Lancet Respir Med 2023; 11: 540–9. 36893780 10.1016/S2213-2600(23)00041-3

[3] Li S, Lv W, Zhou K, et al. Does the fissureless technique decrease the incidence of prolonged air leak after pulmonary lobectomy? Interact Cardiovasc Thorac Surg 2017; 25: 122–4. 28379438 10.1093/icvts/ivx061

[4] Gómez-Caro A, Calvo MJ, Lanzas JT, et al. The approach of fused fissures with fissureless technique decreases the incidence of persistent air leak after lobectomy. Eur J Cardiothorac Surg 2007; 31: 203–8. 17175163 10.1016/j.ejcts.2006.11.030

[5] Temes RT, Willms CD, Endara SA, et al. Fissureless lobectomy. Ann Thorac Surg 1998; 65: 282–4. 9456145 10.1016/s0003-4975(97)01268-x

[6] Igai H, Kamiyoshihara M, Numajiri K, et al. Feasibility and safety of uniportal thoracoscopic segmentectomy using a unidirectional dissection approach without dissecting a fissure. Medicina (Kaunas) 2024; 60: 994. 38929611 10.3390/medicina60060994 PMC11205414

[7] Gonzalez D, Paradela M, Garcia J, et al. Single-port video-assisted thoracoscopic lobectomy. Interact Cardiovasc Thorac Surg 2011; 12: 514–5. 21131682 10.1510/icvts.2010.256222

[8] Akiba T, Nakada T, Inagaki T. Simulation of the fissureless technique for thoracoscopic segmentectomy using rapid prototyping. Ann Thorac Cardiovasc Surg 2015; 21: 84–6. 24633132 10.5761/atcs.nm.13-00322 PMC4989993

[9] Liu C, Liao H, Guo C, et al. Single-direction thoracoscopic basal segmentectomy. J Thorac Cardiovasc Surg 2020; 160: 1586–94. 32111428 10.1016/j.jtcvs.2020.01.028

[10] Sueyoshi K, Kojima F, Otsubo K, et al. Single-direction approach for thoracoscopic segmentectomy of the left upper lobe anterior segment with mediastinal lingular artery. Innovations (Phila) 2022; 17: 156–8. 35323057 10.1177/15569845221086561

[11] Matsui Y, Oizumi H, Watanabe H, et al. Thoracoscopic posterior approach for an S6 left lower lobe segmentectomy in a patient with an incomplete fissure: A case report. J Pediatr Surg Case Rep 2024; 104: 102809.

[12] Han DP, Chen K, Zhang YJ, et al. Uniportal video-assisted thoracoscopic combined segmentectomy for lung cancer with incomplete fissure. J Thorac Dis 2017; 9: 1140–3. 28523171 10.21037/jtd.2017.03.78 PMC5418288

[13] González-Rivas D, Lirio F, Sesma J. Uniportal anatomic combined unusual segmentectomies. J Vis Surg 2017; 3: 91. 29078653 10.21037/jovs.2017.05.12 PMC5637539

[14] Suda T, Nagano H, Negi T, et al. Lateral approach using grasping technic for uniportal major lung resection. Gen Thorac Cardiovasc Surg 2022; 70: 104–6. 34545464 10.1007/s11748-021-01706-1

[15] Kara HV, Balderson SS, D’Amico TA. Modified uniportal video-assisted thoracoscopic surgery (VATS). Ann Cardiothorac Surg 2016; 5: 123–6. 27134839 10.21037/acs.2016.03.09 PMC4827406

[16] Taguchi S, Saeki N, Morio A, et al. Novel technique for identification of the pulmonary intersegmental plane using manual jet ventilation during pulmonary segmentectomy. Wideochir Inne Tech Maloinwazyjne 2021; 16: 169–74. 33786131 10.5114/wiitm.2020.95919 PMC7991954

